# Advances in Anthelmintic Target Identification

**DOI:** 10.3390/ijms26083738

**Published:** 2025-04-15

**Authors:** Harrison T. Shanley, Tao Wang, Aya C. Taki, Joseph J. Byrne, Bill C. H. Chang, Brad E. Sleebs, Robin B. Gasser

**Affiliations:** 1 Department of Veterinary Biosciences, Melbourne Veterinary School, Faculty of Science, The University of Melbourne, Parkville, VIC 3010, Australia; htshanley@gmail.com (H.T.S.); tao.wang1@unimelb.edu.au (T.W.); aya.taki@unimelb.edu.au (A.C.T.); byrnej1@unimelb.edu.au (J.J.B.); bchang@ozomics.com (B.C.H.C.); 2 Walter and Eliza Hall Institute of Medical Research, Parkville, VIC 3052, Australia

**Keywords:** anthelmintic target identification, nematocide, *Haemonchus contortus*, *Caenorhabditis elegans*

## Abstract

Parasitic nematodes pose a significant threat to human and animal health, causing widespread morbidity and substantial socioeconomic losses globally. Despite the utility of anthelmintic drugs in parasite control, the emergence of widespread resistance necessitates the discovery of novel interventions. Advances through the use of whole-organism phenotypic screening have identified some promising nematocidal compounds, including nemacol, tolfenpyrad, UMW-9729, and ABX464. This article summarises efforts in this discovery, with a focus on *Haemonchus contortus* and *Caenorhabditis elegans* as model nematodes, and discusses approaches used for drug target deconvolution, including proteomic, chemical and genetic/genomic techniques. Stability-based proteomic assays, such as thermal proteome profiling, have been useful for identifying protein targets for these compounds, shedding light on their mechanisms of action. However, challenges remain in extrapolating findings from *C. elegans* to parasitic nematodes, emphasising the need for validation studies. Understanding drug–target interactions in nematodes is critical for developing next-generation anthelmintics and for mitigating the growing resistance challenge. This review outlines recent progress in this area and discusses future directions in target validation and anthelmintic development to support parasite control programmes.

## 1. Importance of Parasitic Nematodes and Impetus for Drug Discovery

Infections and diseases (helminthiases) caused by parasitic worms (helminths) have an adverse impact on the health of animals—including humans—and plants, inflicting substantial socioeconomic losses globally [1,2,3,4,5,6]. In 2019, ~909 million people were estimated to be infected with intestinal nematodes (such as *Ascaris*, *Trichuris*, *Ancylostoma,* and *Necator*) [7]. Meanwhile, strongylid nematodes, such as *Haemonchus*, *Ostertagia*, *Teladorsagia*, *Trichostrongylus* and *Cooperia*, contribute to annual losses of USD 2.4 billion to the livestock animal industries in Australia and Europe, with the global market for antiparasitic drugs (anthelmintics) accounting for ~USD 8 billion [5,8,9]. Worm control relies on chemotherapeutic and non-chemotherapeutic methods, and often, multiple strategies are used to meet an individual farm’s needs [10].

**Figure 1:**
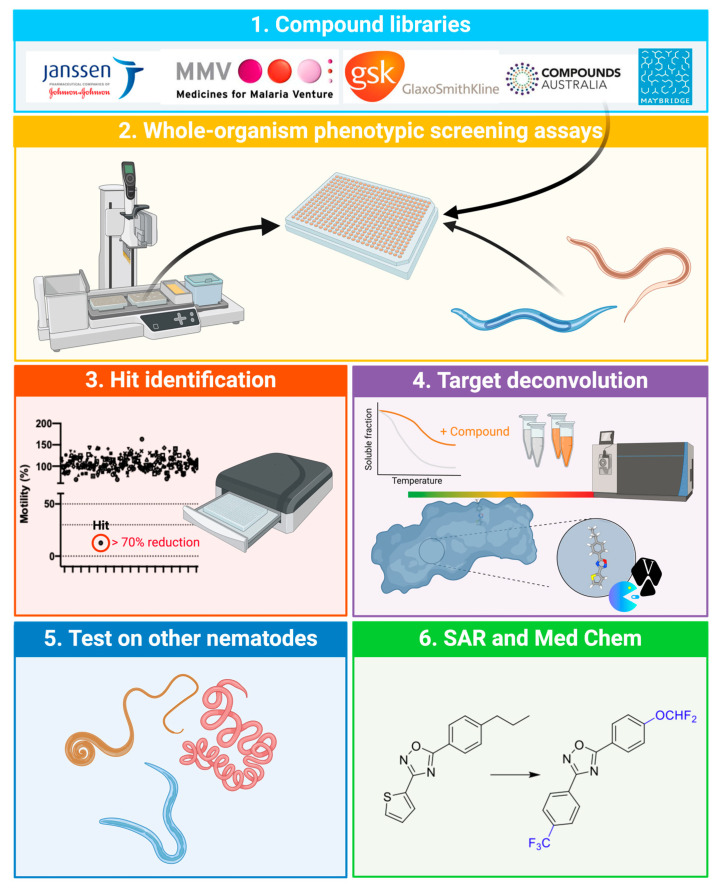
Example of a workflow used for anthelmintic discovery, including six steps (cf. [21]): First, curated libraries of compounds are selected. Second, the libraries are screened individually for in vitro activity in the larvae of the nematodes *Haemonchus contortus* (parasitic) or *Caenorhabditis elegans* (free-living) in established whole-organism, phenotypic screening assays. Third, following incubation with compounds, the effect on phenotype (e.g., motility) is measured using an automated system and test compounds with an anthelmintic effect (i.e., ‘hits’) are identified. Fourth, a method for target deconvolution (e.g., thermal protein profiling) is used to explore compound–target interactions and infer target(s). Molecular docking (e.g., using AutoDock) of individual candidate compounds into their potential binding site(s) within the inferred target is studied in silico. Fifth, hit compounds are assessed for in vitro activity in other parasitic nematodes (including *Heligmosmoides polygyrus*, *Necator americanus* and *Trichuris muris*). Sixth, the activity and potency of compounds are optimised by medicinal chemistry and structure–activity relationship (SAR) studies.

Anthelmintics have been an effective way of suppressing parasitic nematode infections in livestock populations [11,12,13]. However, the uncontrolled and often excessive usage of anthelmintics has led to widespread resistance in parasite populations to most commercially available compounds [12,14,15]. Given that many parasite control programmes still rely heavily on anthelmintics, there is a need to discover new interventions. Recently, drug discovery efforts [16,17,18] (cf. Figure 1) utilising whole-organism, phenotypic screening platforms have identified compounds, such as tolfenpyrad [16], nementin [19], nemacol [20], UMW-878 [21], UMW-9729 [22], and ABX464 [23,24,25], with anthelmintic activity against multiple nematode species, providing the prospects for new nematocides. These projects have largely focused on the use of two key nematode organisms as models for anthelmintic drug discovery: *Haemonchus contortus*, a highly pathogenic, blood-feeding nematode of small ruminants, and *Caenorhabditis elegans*, a related but free-living nematode.

A critical aspect of drug development is the elucidation of the mechanisms by which anthelmintic activity is achieved. Prospective target deconvolution has been complemented by advances in the field of multi-omics of nematodes including *C. elegans* and *H. contortus* [26,27] as well as developments in laboratory-based, target identification techniques. For instance, proteomic approaches, such as the use of stability-based assays [21] and genomic/genetic methods [28], have proven to be successful. The use of *C. elegans* as a comparator model has been particularly instrumental in the target deconvolution of several important anthelmintic drug classes, e.g., levamisole [29], ivermectin [30] and monepantel [31]. Utilising functional genomic, transcriptomic and/or proteomic studies to identify novel mechanism(s) of action has also become an integral step in evaluating novel anthelmintic compounds (cf. [32,33,34,35]).

This article reviews recent efforts to deconvolute nematocide targets, focusing on summarising proteomic, chemical and genetic/genomic approaches for the inference or identification of drug targets in nematodes, and discusses likely challenges associated with using *C. elegans* as a model for target discovery in parasitic nematodes.

## 2. Tools for Drug Target Deconvolution

Target identification provides a path to infer the mechanism of action of an active compound and can be conducted using proteomic, chemical, and/or genomic approaches (Table 1).

### 2.1. Proteomic Approaches

Stability-based methods, whereby changes in the biophysical characteristics of a protein upon incubation with a bioactive compound are measured, offer a means of target identification without the need for compound modification [21,36,37,38,39,40,41,42]. Notable examples of stability-based target identification include drug affinity responsive target stability (DARTS), thermal proteome profiling (TPP), and cellular thermal shift assay (CETSA).

Drug affinity responsive target stability (DARTS) relies on the principle that protein–drug bound complexes are less susceptible to proteolytic degradation [36,37]. Thus, a protein lysate can be incubated with a bioactive compound; the mixture can then be exposed to a protease solution; differentially proteolysed target proteins, in the presence/absence of a drug, can then be analysed via polyacrylamide gel electrophoresis (SDS-PAGE), Western blotting, or more advanced MS-based proteomics [37]. Recently, Zhang et al. [43] used a DARTS assay to identify several *C. elegans* mitochondrial protein targets of epigallocatechin gallate and theaflavin; however, the use of this assay in a parasitic nematode model has not yet been reported.

TPP is a proteome-wide, mass spectrometry-based method for unbiased target discovery, detecting both direct and indirect interactions, whereas the cellular thermal shift assay (CETSA) is a targeted, lower-throughput approach, typically using Western blot or proteomics to validate known or suspected interactions. TPP is ideal for broad discovery, and CETSA is better suited for confirming specific targets. These thermal stability methods exploit the stabilisation of proteins when bound to a compound [21,39,40,41,42]. Upon increased temperature challenge, proteins will begin to unfold and precipitate from a solution; however, stabilised proteins will be less susceptible to degradation and will remain in solution. Hence, drug–protein interactions can be identified by comparing the stability of proteins in the presence/absence of a compound over a range of temperatures, or alternatively, at a single temperature over a range of drug concentrations [21,39,40,41,42]. TPP and CETSA allow for a holistic approach to identifying drug–protein (including off-target) interactions, illuminating several possible target(s). Indeed, by exploiting protein thermostability [44] to gauge drug–protein interactions, TPP and CETSA can offer advantages over functional genomics methods, which can be laborious, costly, and are not yet well-established for parasitic nematodes.

TPP was applied recently, for the first time, to a parasitic nematode [21], identifying two possible protein targets (designated as HCON_014287 and HCON_011565) of an anthelmintic candidate, UMW-868, in *H. contortus* larvae. This established platform was then applied to two other unique candidates, ABX464 and UMW-9729 [22,24,25] on protein lysates of both *H. contortus* and *C. elegans*. For ABX464, one *H. contortus* protein (designated as HCON_00074590) and four *C. elegans* proteins (CRN-3, F30F8.9, RAGA-1 and NKCC-1) were found to be significantly stabilised in the presence of ABX464, indicative of compound–protein binding. For UMW-9729, three *H. contortus* proteins and five “conditionally essential” *C. elegans* gene products (cf. [45]) were significantly stabilised in the presence of this compound; however, these proteins were not found to be structurally or functionally related to each other, suggesting that this compound does not target a conserved nematode protein. This proposal warrants experimental testing using complementary and/or orthogonal validation techniques to establish whether UMW-9729 does indeed interact with different proteins in distinct species.

These are early efforts at applying TPP to a parasitic nematode (*H. contortus*), giving insights into the considerations, challenges, and opportunities for protein-focused target identification and validation. Employing TPP in the comparator model, *C. elegans*, provides a possible pathway to identifying one or more conserved protein target(s). However, several considerations should be made regarding the methodology and interpretation of results when performing TPP. For instance, if the primary target protein of a compound is not significantly denatured in an increasing temperature gradient, then a genuine target may not be identified [39,40,41,42]. Moreover, these methods are prone to false-positive results; hence, the use of suitable control samples is necessary for robust target identification. In the work performed by Taki et al. [21] and Shanley et al. [22,24,25], the stability of proteins in the presence of test compounds (UMW-9729 or ABX464) was referenced to a non-treated control. Whilst reasonable, this method could be enhanced through the inclusion of an inactive test analogue to identify possible ‘off-targets’ and potentially a positive control with a known protein target, such as monepantel, which could assess the performance of the assay and accuracy of the results. Moreover, the work here focused predominantly on proteins stabilised by compound binding and did not comprehensively explore proteins that were destabilised. This could reflect a compound-induced disruption of protein complexes important to worm survival and, thus, should be considered in future deconvolution/validation studies. Enhancing the TPP workflow, according to the changes mentioned here, could strengthen proteomic-based target deconvolution in parasites and might be considered in future studies.

Another method that shows promise for identifying nematocide targets and has been recently applied to the malaria parasite, *Plasmodium falciparum*, is integral solvent-induced protein precipitation (iSPP) [46,47]. This technique allowed for the assessment of protein stability in the presence of antimalarial compounds, facilitating the identification of potential drug targets within the parasite’s proteome. Additionally, chemoproteomic approaches based on thermal stability and limited proteolysis have been employed to study target engagement in parasites. These methods have been used to validate the on-target activity of specific inhibitors in *P. falciparum*, providing insights into the drug’s mechanism of action and aiding in the development of new antiparasitic therapies [48]. These applications demonstrate the utility of solvent-induced proteome profiling and related techniques in advancing our understanding of host–parasite interactions and in the discovery of novel therapeutic targets against parasitic diseases.

### 2.2. Chemical Probe-Based Approaches

Affinity- or activity-based ‘pull-down’ assays, using chemical probes, are widely used for target identification. In principle, a compound of interest (in this case, an anthelmintic) is attached to a solid support or functional tag, and in the case of activity-based protein profiling, is also conjugated to a reactive warhead [38,42]. The conjugated compound is then incubated with a protein lysate; the compound will either intrinsically bind with protein targets (affinity-based) or irreversibly interact with protein target residues (activity-based). After washing or eluting with unlabelled compound to remove non-specifically bound proteins, the targets can be identified via SDS-PAGE and characterised using MS analysis [38,42,49].

Him et al. [50] used an affinity-based assay to investigate the binding of both *C. elegans* and *H. contortus* (amongst several other nematode species)-derived heat shock protein 90 (HSP-90), essential for protein chaperoning, and an inhibitor of HSP-90, geldanamycin (GA) [50]. Here, GA (modified with 1,6-hexanediamine) was attached to a solid support (Affi-Beads); worm lysates were prepared and mixed with the GA–solid support beads; and the beads were then washed with the GA-bound proteins analysed via SDS-PAGE and Western blotting [50]. It was identified that in nematodes with free-living larval life stages (such as *C. elegans* and *H. contortus*), GA did not bind to HSP-90, yet in obligate parasites (such as filarial nematodes), GA-HSP-90 binding was identified, a result which correlated with the differential lethality displayed by GA treatment of nematode species.

Although an effective means of target identification, several hurdles in chemical probe-based target identification exist. Chemical modification of a hit compound for matrix linkage, without disrupting activity/affinity, can require a significant amount of time and effort [42]. One such way to circumvent this issue is through photo-affinity labelling [51]. Here, a photo-affinity probe is first formed via the attachment of a bioactive compound to a photoreactive moiety via a linker [51]. Photoreactive moieties can generate reactive diradicals (from benzophenones), carbenes (from diazirine), or nitrenes (from aryl azides) upon excitement with UV light; these reactive groups can then cross-link to adjacent proteins [51]. Thus, photo-affinity probes can be incubated with a protein lysate and exposed to UV light, and (after extensive washing) drug–protein interactions can be identified. However, as with affinity- and activity-based binding, non-specific binding can obscure the detection of genuine targets; as such, adequate negative and positive controls are crucial for the identification of genuine targets [38,42].

There is ample opportunity to extend other protein-based assays, such as isothermal dose–response fingerprinting [39], proteome integral solubility alteration (PISA; [52]), or affinity-based assays, to a parasitic nematode model, such as *H. contortus*. Indeed, the successful implementation of such methods into anthelmintic discovery programmes would greatly advance the development of novel antiparasitics to control helminth diseases.

### 2.3. Genetic Approaches

An alternative method for identifying direct drug–protein interactions is to search for genetic changes that confer phenotypic resistance/sensitivity to drug effects. Here, three approaches—resistance studies, RNA interference (RNAi) and CRISPR/Cas9 technology—are discussed.

#### 2.3.1. Resistance Assays

*C. elegans* is highly amenable to drug target identification in resistance assays [53,54,55,56]. Most often, wild-type *C. elegans* worms are treated with a chemical mutagen (e.g., ethyl methane sulfonate, trimethylpsoralen, and short-wave UV) [54,56]. Progeny can then be screened for individuals that are resistant to a molecule’s effects [54,56]; the genome of resistant populations can then be mapped to identify polymorphisms in genes that may confer drug resistance, allowing for the identification of drug targets. Burns et al. [54] identified 15 mutants (of 180,000 mutagenised genomes) that conferred resistance in *C. elegans* to the molecule nemadipine-A [57], 14 of which were in *egl-19*, a known target of nemadipine-A. This powerful technique has also been used to identify the targets of levamisole [29], ivermectin [30] and monepantel [31].

Producing drug-resistant worms via chemical mutagenesis is less practical in a parasitic worm, given that the life cycle is dependent on a host animal. However, resistance can be induced via repeated drug treatment, albeit laborious and time-consuming [31]. Kaminsky et al. [31] identified the drug target of monepantel (DES-2) in *H. contortus* via a resistance study. Here, *H. contortus* eggs were first allowed to develop into L3s under drug pressure, with surviving larvae then used to re-infect sheep, with monepantel resistance being achieved after eight such cycles [31]. Whilst effective, given the length of time necessary for *H. contortus* larvae to develop into dioecious adults within a host, this procedure could take years to complete, depending on how rapidly resistance is conferred. Thus, the use of the free-living nematode *C. elegans*, in which a resistance study might only take months, might be preferred over the use of a parasitic species, on the condition that anthelmintic compounds share orthologous targets across both species.

#### 2.3.2. RNA Interference (RNAi)

Comparative to resistance studies, RNAi employs a reverse genetics approach [58,59,60]. Several double-stranded RNA (dsRNA) libraries have been synthesised, which represent most genes encoded in the *C. elegans* genome [53,61]. RNAi-mediated knockdown of genes in *C. elegans* is achieved via the induction of dsRNA into the worm [58,61]. *C. elegans-* knockdown mutants can then be analysed for phenotypic responses. For example, a genome-wide RNAi analysis of fat regulatory genes in *C. elegans* was able to identify 305 gene inactivations that cause reduced body fat as well as 112 gene inactivations that cause increased fat storage [62]. Many of the identified fat regulatory genes had mammalian homologues, representing an exciting step for identifying protein targets to treat human obesity [62].

RNAi has also been used with some degree of success in parasitic species, such as *H. contortus* (reviewed in [63]). Intriguingly, Blanchard et al. [64] used RNAi to demonstrate that the gene silencing of *Hc-acr-8* reduced the sensitivity of *H. contortus* larvae to levamisole. Importantly, *Hc-acr-8* presented as a functional substitute for *Ce-lev-8* (a gene encoding levamisole-sensitive AChR in *C. elegans*), highlighting a divergence between a parasitic and free-living species. However, compared with *C. elegans*, RNAi in *H. contortus* (and other strongylids) has been challenging [59,60,63,65]. *H. contortus* RNAi sensitivity varies based on dsRNA delivery (immersion, feeding or injection) and has issues with reproducibility. Moreover, most of the work has been conducted on *H. contortus* L3s, making assessment of phenotypes expressed in later development stages difficult [63].

#### 2.3.3. CRISPR/Cas9

An alternative method for genome engineering is the CRISPR (clustered regularly interspaced palindromic repeat)/Cas (CRISPR-associated nuclease) 9 system [66]. CRISPR/Cas-9 technology provides a specific, direct knockout of target genes, overcoming the limitations of RNAi. In principle, an endonuclease (such as Cas9) is attached to a single guide RNA sequence (sgRNA); the sgRNA directs the nuclease to a target DNA; and the nuclease will then create a double-stranded break in the DNA, allowing for either gene ‘knock-out’ or ‘knock-in’ [66]. The CRISPR/Cas9 system has been successfully used in *C. elegans* [67,68], as well as in parasitic nematodes such as *S. stercoralis*, *Brugia malayi* and *Ni. brasiliensis* [28,69,70,71]. In *S. stercoralis*, gonadal microinjection of CRISPR/Cas9 constructs (targeting *Ss-unc-22*) into free-living adults resulted in severe motility defects in F_1_ infective L3 progeny [69]. The mutant worm progeny was also recoverable after being passed through a host species [69]. Liu et al. [70] demonstrated that lipofection of L3s of *Br. malayi* with a CRIPSR/Cas9 construct and subsequent infection of a host species with transfected L3s produced transgenic F_1_ microfilariae; however, only ~3% of the recovered progeny were found to be transgenic. CRISPR/Cas9-mediated editing of the DNase II gene was also used in *Ni. brasiliensis*, delivered to L3s via extracellular vesicles [71]. Despite the need for some refinement, these studies are evidence that extending CRISPR technology to other parasitic nematodes, such as *H. contortus*, could be possible and could significantly aid drug target identification efforts. For instance, the effect of a small molecule on worms in which a given gene has been repressed, induced or deleted (via CRISPR), compared with a wild-type organism, could give insight into how anthelmintic activity is achieved [72]. Although not yet reported for *H. contortus*, the development of CRISPR technology for use in parasitic helminths offers many opportunities for future drug discovery efforts, providing a robust method for genome editing.

## 3. Challenges Associated with Using *C. elegans* as a Model for Nematocide Discovery and Target Deconvolution

It has been proposed that using two genetically related yet biologically distinct nematode species—such as *H. contortus* and *C. elegans*—could enable the identification of a broad-spectrum anthelmintic with a unique, conserved nematode-specific mechanism of action. Moreover, although outside the scope of this work, the amenability of *C. elegans* to genetic manipulation could enable orthogonal approaches to drug target identification and validation. However, recent investigations of novel candidate drug targets in *H. contortus* and *C. elegans* have given some insights into the utility and limitations of *C. elegans* as a comparator model for *H. contortus*.

Indeed, in the previously-discussed TPP studies of the novel scaffolds ABX464 and UMW-9729 in *H. contortus* and *C. elegans* [22,24,25], a conserved nematode protein target was not identified between the two species. It could be that the anthelmintic action of each compound was being achieved in both organisms via unique drug–target interactions, testing the usefulness of *C. elegans* as a comparator. It is also plausible that (in both instances) TPP was unable to identify the genuine nematode protein targets of either chemical entity (either proteins were not significantly denatured, or compound-induced stabilisation was not sufficiently reached). Complementary and/or validation of the drug–protein interactions identified here could elucidate why a conserved nematode protein target was not identified. However, in the case that the test compounds are not targeting proteins common to both species, it may be that *C. elegans* is not an optimal comparator for *H. contortus*.

Promisingly, however, in the case of ABX464, moderate broad-spectrum activity against several parasitic nematodes was found. Furthermore, it has been previously demonstrated that many of the commercially available anthelmintics do indeed target similar proteins across several important species, including *H. contortus* and *C. elegans* [73]. Contemporary studies, such as those undertaken by Burns et al. [74] and Harrington et al. [19,20], demonstrate that novel anthelmintic compounds (designated ‘nemacol’) first identified in *C. elegans* do, indeed, display activity in related parasitic nematodes, such as *H. contortus*. From this work, it is evident that *C. elegans* remains a useful model organism for anthelmintic discovery; however, it is a comparator or complementary tool rather than a substitute.

## 4. Concluding Remarks

This review highlights recent efforts in novel nematocide discovery, including the early-stage identification of compounds such as UMW-878, ABX464, and UMW-9729 (e.g., [21,22,23,24,25]). We also discussed proteomic- and genetic-based tools for drug target deconvolution. However, many of these approaches have yet to be applied to molecularly well-characterised parasitic nematode models, such as *H. contortus*. While *C. elegans* has been widely used for target identification, relying on a free-living model may lead to misleading conclusions.

A greater focus on mechanism-of-action studies in *H. contortus* (cf. [75]) could significantly improve discovery efforts by providing a more accurate assessment of nematocidal and nematostatic activity. Crucially, protein candidates identified through target identification methods must be rigorously validated, where possible, using reverse genetics (e.g., RNAi knockdown or CRISPR-mediated knockout), recombinant protein expression with biophysical binding assays, and/or complementary target identification techniques. Integrating these approaches into early-stage drug development would strengthen confidence in target engagement, reveal new opportunities for anthelmintic intervention and provide deeper insights into resistance mechanisms. These advances will be critical for developing effective nematocides to support parasite control, particularly in cases where resistance to all existing anthelmintic classes has emerged.

## Figures and Tables

**Table 1:** Summary of some key techniques used for drug target deconvolution.

Category	Techniques	Principle	Advantages	Disadvantages
(i) Proteomic approaches				
	DARTS (drug affinity responsive target stability)	Measures protein stability upon drug binding via proteolysis.	No need to modify compounds (label-free); simple setup.	Requires optimisation; prone to false positives; does not work on protease-resistant proteins.
	Thermal shift assays: CETSA (cellular thermal shift assay) and TPP (thermal proteome profiling)	Monitors drug-induced thermal stability of proteins.	Label-free; applicable to lysed or live cells; high-throughput potential.	Need high concentrations of compound; may not detect weak binders; data analysis is complex. Prone to detecting binding of non-essential proteins.
	SPROX	Uses methionine oxidation to assess drug–protein interactions.	Label-free; unbiased and quantitative; proteome-wide applications.	Only proteins/binding sites with methionine residues are detected. Met oxidation can be heterogeneous, complicating analysis.
	iSPP (integral solvent-induced protein precipitation)	Monitors solvent-induced precipitation to detect drug–protein binding.	Label-free; can detect novel interactions; useful for parasites.	Requires careful validation; limited broad application.
(ii) Chemical probe-based approaches				
	Affinity purification (pull-down)	Uses modified/labelled compounds to pull down binding proteins.	Directly isolates genuine binding partners; well-established.	Need compound SAR to apply chemical label. Label prone to false positives unless stringent compound and biological controls are used. Only works on parasite lysate.
	Photoaffinity labelling	Uses UV-activated probes to covalently bind targets.	Captures transient interactions; applicable to live cells.	Needs SAR to label compound; UV irradiation can cause non-specific binding; requires irradiation equipment.
	Activity-based protein profiling (ABPP)	Uses covalent probes to label active-site residues of enzymes.	High specificity for enzyme targets; detects active proteins.	Limited to enzymes; requires designing reactive and selective probes.
(iii) Genetic approaches				
	Resistance assays	Identifies drug targets by analysing resistance mutations.	Unbiased; powerful for finding essential drug pathways.	Labour-intensive; typically requires chemically induced mutagenesis; mutations can be indirect or a resistance mechanism, e.g., drug efflux pump.
	RNA interference (RNAi)	Uses dsRNA to silence genes and assess drug effects.	Efficient in *C. elegans*; high-throughput screens possible.	Limited effectiveness with RNAi uptake in some parasitic nematodes.
	CRISPR/Cas9	Uses gene editing to knock out or modify target genes.	Highly specific; enables functional validation.	Requires development and optimisation for most parasites.
